# Focal Pulsed Field Ablation for Atrial Arrhythmias: Efficacy and Safety under Deep Sedation

**DOI:** 10.3390/jcm13020576

**Published:** 2024-01-19

**Authors:** Sebastian Weyand, Viola Adam, Paloma Biehler, Patricia Hägele, Simon Hanger, David Heinzmann, Stephanie Löbig, Andrei Pinchuk, Christian Waechter, Peter Seizer

**Affiliations:** 1 Department of Cardiology, Ostalb Clinic Aalen, Im Kaelblesrain 1, 73430 Aalen, Germany; sebastian.weyand@kliniken-ostalb.de (S.W.); viola.adam@kliniken-ostalb.de (V.A.); paloma.biehler@kliniken-ostalb.de (P.B.); patricia.haegele@kliniken-ostalb.de (P.H.); simon.hanger@kliniken-ostalb.de (S.H.); stephanie.loebig@kliniken-ostalb.de (S.L.); andrei.pinchuk@kliniken-ostalb.de (A.P.); 2 Department of Cardiology, University Hospital Tuebingen, Otfried-Mueller-Straße 10, 72076 Tuebingen, Germany; 3 Department of Cardiology, University Hospital Marburg, Philipps University Marburg, Baldingerstraße, 35043 Marburg, Germany; christian.waechter@uni-marburg.de

**Keywords:** atrial fibrillation, atypical flutter, repeat ablation, CENTAURI, coronary vasospasm, adenosine testing

## Abstract

Focal pulsed field ablation (PFA) is a novel technique for treating cardiac arrhythmias. It has demonstrated positive results in initial studies and has a good safety profile. In recent studies, PFA was often utilized for first-time pulmonary vein isolation (PVI) and was performed under general anesthesia. In our study, we assessed the feasibility, safety, acute procedural efficacy, and efficiency of focal PFA under deep sedation in patients, 80% of whom had undergone at least one left atrial ablation previously. We treated 30 patients (71 ± 7, 46% male) using the CENTAURI system for various atrial arrhythmias, including atrial fibrillation, typical and atypical atrial flutter, and focal atrial tachycardia. The average procedure and fluoroscopy times were 122 ± 43 min and 9 ± 7 min, respectively. A total of 83.33% of patients received additional line ablations beyond PVI, specifically targeting the posterior box and anterior mitral line. All ablations were successfully performed in deep sedation with only one major and one minor complication observed. The major complication was a vasospasm of the right coronary artery during ablation of the cavotricuspid isthmus, which was treated successfully with intracoronary nitroglycerin. All patients could be discharged in sinus rhythm. Moreover, adenosine appears effective in identifying dormant conduction in some patients after focal PFA. In conclusion, focal PFA is an effective approach for complex left atrial ablations under deep sedation, offering both high efficacy and efficiency with a reliable safety profile. Studies on long-term outcomes are needed.

## 1. Introduction

The prevalence of atrial fibrillation (AF), which is the most frequently occurring arrhythmia worldwide, is significant, affecting 22–26% of the population at some point in their lives, and it thus has considerable implications for public health [1]. Studies have demonstrated that AF catheter ablation, in contrast to just medical treatment, can be a successful intervention, enhancing the quality of life for patients [2,3]. In addition to the long-established procedures for pulmonary vein isolation (PVI) using radiofrequency or cryoenergy, another form of energy, pulsed field ablation (PFA), has recently been introduced. This innovative technique utilizes irreversible electroporation to create efficient and profound lesions [4]. Furthermore, it can be optimized for maximum selectivity towards cardiac cells, thereby minimizing the risk of injury to extracardiac structures such as the esophagus or phrenic nerve [5,6]. After promising prospective data on three different single-shot devices [7,8,9], the first system for focal PFA, the CENTAURI System (Galaxy Medical, San Carlos, CA, USA), has received CE certification in the European Union. This certification indicates compliance with EU safety, health, and environmental protection standards, allowing its use within the EU member states. Unlike single-shot PFA devices, which are primarily utilized for sole PVI, the CENTAURI System’s technology, based on focal point-by-point ablation, also enables the creation of additional ablation lines. This system consists of a generator that can be coupled with various ablation catheters and mapping systems. While successful ablation of both atrial [10] and ventricular arrhythmias [11] has been demonstrated using this system, numerous questions remain regarding its optimal use, appropriate sedation strategies, and the determination of reliable ablation endpoints.

## 2. Materials and Methods

### 2.1. Patient Selection

This retrospective study included 30 adult patients with symptomatic AF or atrial tachycardia (AT) undergoing focal PFA with the CENTAURI system in Ostalb-Klinikum Aalen from 1 January 2023 to 18 July 2023. The procedures were consistently performed by one of two experienced electrophysiologists from the center. There were no specific exclusion criteria in this study, and every patient treated with PFA in this time period was included. The final decision to use catheter-based cardiac irreversible electroporation was at the discretion of the electrophysiologist, who performed the procedure and typically opted for patients with previous atrial fibrillation ablations or those who specifically requested this energy form. All patients underwent their ablation due to recurrent symptomatic episodes of arrhythmia. This study was approved by the ethics committee of the Landesärztekammer Baden-Württemberg. All patients gave informed consent to the ablation procedure and post ablation diagnostics.

### 2.2. Ablation Procedure

**Figure 1:**
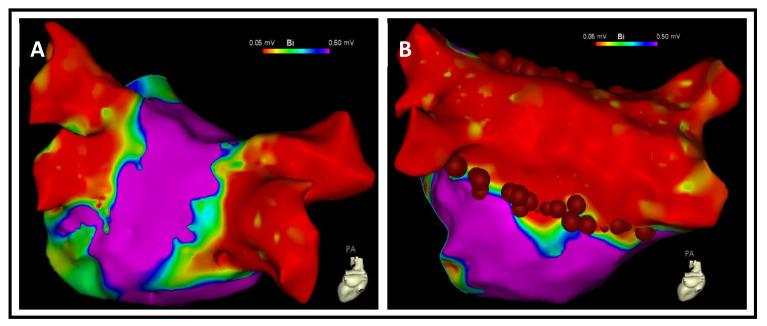
Example of left atrial posterior wall isolation and lateral mitral isthmus ablation in a patient with two prior PVIs and durably isolated pulmonary veins. Panel (**A**) displays the bipolar voltage map pre-ablation, while Panel (**B**) illustrates the post-ablation voltage map. Red tags denote the PFA application sites. Voltage maps are color-coded: purple represents local electrograms > 0.5 mV, and red indicates local electrograms < 0.05 mV.

Transesophageal echocardiography was performed before the procedure. All patients were anticoagulated at least 3 weeks before and 3 months after the procedure independent of CHA_2_DS_2_-VASc score. Patients were anticoagulated with intravenous heparin during the procedure. The infusion was adjusted to maintain an activated clotting time of 300–400 s. Patients were safely positioned and secured using gentle restraints on both their forearms and thighs throughout the procedure. Detailed electroanatomic data were obtained using either the CARTO 3 System Version 7 (Biosense Webster, Diamond Bar, CA, USA) or Ensite X Software Version 3 (Abbott Laboratories, Chicago, IL, USA) mapping system. Double transseptal access was obtained. The high-resolution mapping catheter (CARTO Pentaray, Biosense Webster/Advisor HD Grid, Abbott Laboratories) and an irrigated and contact force-sensing ablation catheter (NAVISTAR SMARTTOUCH, Biosense Webster/TACTICATH QUARTZ, Abbott Laboratories) were inserted into the left atrium (LA) via transseptal sheaths. In all patients, a high-density electroanatomical map of the LA and pulmonary veins (PV) was obtained to visualize anatomy. The extent of low voltage areas (<0.5 mV) in the LA was assessed using the embedded area measurement tools, with the percentage of these areas being calculated in relation to the total surface area of the left atrium. Mapping was carried out in sinus rhythm by default. If the patient was initially in atrial fibrillation, a DC cardioversion was performed. If the patient was in atypical atrial flutter, cardioversion was not performed and local activation time (LAT) mapping was performed. The CENTAURI generator was connected to either the CARTO or Ensite X 3D mapping system [12]. Ablation applications were ECG R-synchronized trains of pulsed field energy with a setting of 25 amperes over 10 pulse trains. The irrigation rate was 4 mL/h. Applications/tags of 6 mm were placed in accordance with the CLOSE protocol [13]. For initial PVI, wide antral circumferential ablation (WACA) was targeted; in case of localized low-voltage substrate, additional lesions were performed. In repeat procedures, the high-resolution map was assessed on isolation, voltage, and scars and all veins were tested for entry and exit block. In case of reconnections of the PV, re-PVI was performed. If all veins were isolated, the ablation strategy consisted of creation of a posterior box (Figure 1) and an anterior mitral line or lateral mitral isthmus ablation. The exact ablation concept was determined individually by the interventionalist based on the substrate in the 3D map and the previous history. Given the known anatomical proximity to coronary arteries at the cavotricuspid isthmus (CTI) and lateral mitral isthmus, we administered a prophylactic intravenous dose of 0.2 mg nitroglycerin prior to initiating pulsed field ablation (PFA) at these sites to prevent coronary artery vasospasm. After ablation, entrance and exit block were demonstrated for each PV and the carinas between PVs and linear anatomical block was demonstrated for additional lines with and without administration of 12 mg of intravenous Adenosine for revealing dormant conduction. Entrance and exit block were checked in the posterior box with a high-resolution mapping catheter, and the anterior mitral line was checked during pacing from the left atrial appendage. Patients stayed in hospital under continuous rhythm monitoring for at least 24 h.

### 2.3. Sedation Protocol

Electrophysiological study ablation was performed in spontaneously breathing patients under intravenous deep sedation. Propofol and remifentanil were infused continuously via a perfusor. The Richmond Agitation-Sedation Scale (RASS) [14] was utilized to determine the infusion rate. Each patient was intended to achieve a RASS of −5 (unarousable; no response to voice or physical stimulation). The operator, a second physician with intensive care expertise, and an intensive care nurse collaboratively assessed sedation levels, adjusting the infusion rates of sedation agents as needed. Before initiating the electroanatomical mapping procedure, a Guedel airway was inserted in each patient. The mapping phase commenced only if the patient showed no response to the insertion of the Guedel airway. When patient agitation or pain or severe cough irritation occurred during ablation, 12.5 mg ketamine was applied. An arterial blood gas analysis was taken in every patient before ablation.

### 2.4. Follow-Up

The follow-up was conducted for 30 days after ablation procedure. Several major and minor complications were evaluated during the study. Major complications included procedure-related deaths, atrio-esophageal fistulae, procedure-related strokes or TIAs, pericardial tamponades requiring intervention, hemothorax, severe air embolism with ST elevation and hemodynamic collapse, new-onset renal failure requiring dialysis, and vascular access complications requiring surgical intervention. Minor complications encompassed vascular access complications that did not necessitate surgical intervention, nitrosensitive ST elevations caused by vasospasm of a coronary vessel under PFA application, acute hypersensitivity reactions to the contrast agent, pneumonia, hemoptysis, postinterventional pericarditis, and small pericardial effusions that resolved spontaneously without intervention. After ablation, each patient was advised to see us immediately if arrhythmias or other symptoms related to AF or heart failure or ablation procedure recur.

### 2.5. Endpoints

The primary procedural endpoint of this study was the acute success of the procedure, characterized by the isolation of all PV, as demonstrated by entry and exit block, and, when applicable, bidirectional linear anatomical block of additional ablation lesions at the end of the procedure. The primary safety endpoint involved assessing the occurrence of predetermined major complications that were relevant to the system and procedure, both during the procedure and within 30 days afterward. Secondary endpoints included procedural parameters such as duration, fluoroscopy time, and dose area product, as well as minor complications. Further details were evaluated concerning deep sedation, including whether intubation became necessary, the occurrence of a mapshift requiring remapping, and any oxygen drop requiring mask ventilation.

### 2.6. Statistical Analysis

Categorical variables are expressed as frequencies and percentages. Continuous variables are presented as mean value ± standard deviation. Data analysis was performed using Excel 2016 (Microsoft Corporation, Redmond, WA, USA) and GraphPad Prism 9 (GraphPad Software, Inc., San Diego, CA, USA). The graphical abstract was created using Mind the Graph (Cactus Communications, Mumbai, India) under a subscriber account.

## 3. Results

### 3.1. Baseline Characteristics

The study consisted of 30 patients (71.07 ± 7.11 years old, 46.47% male) undergoing focal PFA with the CENTAURI system for various types of left atrial arrhythmias. The average body mass index (BMI) of the patients was 27.95 ± 5.03 kg/m^2^. The types of arrhythmias included Paroxysmal AF (10%), Persistent AF (73.33%), and Atrial flutter/ta-chycardia (56.67%). The patients had an average CHA2DS2-VASc score of 2.62 ± 1.21, indicating a moderate stroke risk. The mean left ventricular ejection fraction was 56.64 ± 6.92%. The mean left atrial fibrosis area was 34.09 ± 36.47%, with 60% of patients presenting with moderate or severely dilated left atrium. Hypertension was prevalent in 80% of the patients, while chronic kidney disease and coronary artery disease were observed in 16.67% and 20%, respectively. Diabetes mellitus was found in 3.33% of patients, and no patient had a prior stroke. All patients were on oral anticoagulants, with 73.33% on beta-blockers and 6.67% on verapamil as their antiarrhythmic therapy; none were on amiodarone. Baseline characteristics are summarized in Table 1.

### 3.2. Procedural Data

Successful ablation, demonstrated by entry and exit block for the PV and by bidirectional linear anatomical block for additional lines, was achieved in all patients, with the procedural duration averaging 122.5 ± 42.85 min. Biosense Webster’s 3D mapping system Carto was utilized in 70% of the cases, while Abbott’s EnSite X was employed in the remaining 30%. The fluoroscopy time and dose area product were 9.14 ± 6.65 min and 654.3 ± 502.6 cGy*cm^2^, respectively. In patients undergoing left atrial ablation for the first time, PVI alone was performed in 33.33%, and additional ablation lesions were placed in 66.67%. For those undergoing repeat ablation, re-PVI was performed in 66.67% of the cases, with additional lesions created in 87.5%. Overall, 66.67% of patients received a posterior box ablation, 50% received an anterior mitral line, and 6.67% underwent a lateral mitral isthmus ablation. Cavotricuspid isthmus (CTI) ablation was performed in 13.33% of cases. Post-adenosine conduction recovery was observed in 6.67% of cases. For all these patients, a complete block was demonstrated following reablation and readministration of adenosine. Procedural parameters are detailed in Table 2.

### 3.3. Sedation Parameters

In all patients, the procedure was performed under deep sedation, and intubation was not necessary for any patient. All 30 patients achieved a RASS score of −5 before the initiation of the mapping phase. The mean propofol infusion rate during ablation was 286.3 ± 66.72 mg/h, and the mean remifentanil infusion rate was 134.66 ± 45.76 µg/h. A single dose of 12.5 mg ketamine was required for 46.67% of the patients during the ablation; in 71.42% of these cases, it was necessary due to pronounced cough irritation, especially during ablation at the LA roof. In 28.57% of cases, ketamine was administered because of persistent pain with agitation, despite already-high doses of propofol and remifentanil. In all of these cases, symptoms improved, and ablation could be successfully continued. Nasal oxygen was administered at an average rate of 3.83 ± 1.56 L/min. Only one case (3.33%) required remapping due to mapshift, which was provoked by sudden onset of coughing of the patient under PFA application before ketamine was administered, and no instances of oxygen drop necessitating mask ventilation were observed. The average pH and pCO_2_ levels before the start of ablation were 7.28 ± 0.05 and 56.59 ± 8.29 mmHg, respectively. Sedation parameters are outlined in Table 3.

### 3.4. Complications

**Figure 2:**
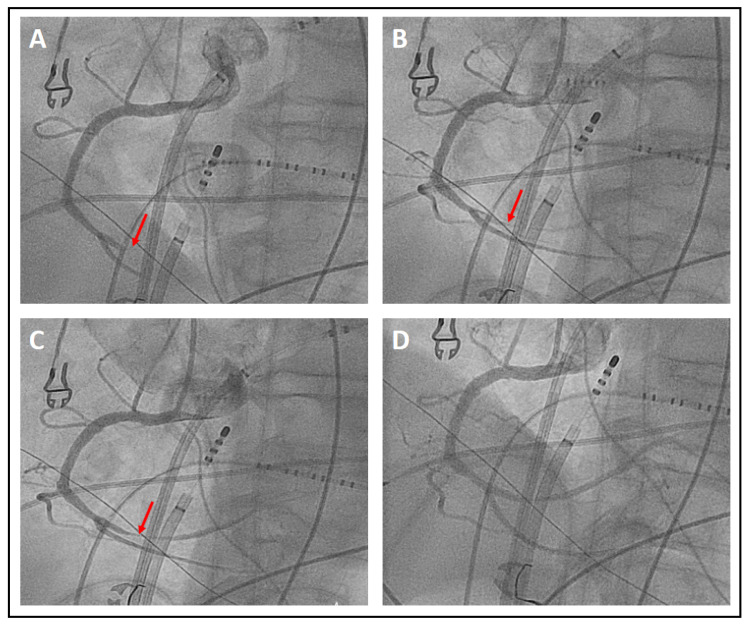
The coronary angiogram depicts a vasospasm of the right coronary artery (arrows) during PFA of the cavotricuspid isthmus. Panel (**A**) shows the coronary angiogram 9 min after the onset of symptoms, followed by Panel (**B**) at 17 min and Panel (**C**) at 18 min, and by the time of Panel (**D**) at 24 min, the vasospasm had nearly completely resolved and symptoms had subsided. During this period, a cumulative dose of 1 mg nitroglycerin was fractionally administered into the right coronary artery.

The study assessed potential major and minor complications in a total of 30 patients during the procedure and within 30 days following the ablation procedure. As major complication, one patient (3.33%) showed ST elevations due to vasospasm of the right coronary artery during PFA application at the CTI. This event was accompanied by temporary hypotension and a third-degree AV-block, which resolved under fractionated intracoronary application of cumulative 1 mg of nitroglycerin (Figure 2). There were no further major complications observed, including procedure-related deaths, atrio-esophageal fistulae, strokes, or tamponades. As for minor complications, one patient (3.33%) experienced prolonged bleeding at the injection site in the right groin, leading to a pronounced hematoma. The overall complication rate was 6.67%. The details of the complications are summarized in Table 4.

## 4. Discussion

This study evaluated the feasibility, safety, and effectiveness of atrial arrhythmia ablation under deep sedation. Patient characteristics were typical of atrial fibrillation cases [15], similar to other focal pulsed field trials using the CENTAURI system [10,16]. In our study, a significant 80% had previous ablations, and 50% presented with atypical flutter or atrial fibrillation at the onset of the procedure. Our results show that even in this patient group, focal PFA is safe and efficient without general anesthesia, achieving 100% acute procedural success.

### 4.1. Procedural Data

Our study confirmed an acute success rate in all cases for isolating PVs and blocked linear lines, aligning with previous high success rates of PFA [17]. Chronic success, as shown by Anić et al., indicated 89% PV isolation at 90 days in patients receiving first PVI with focal PFA [16]. Future studies with extended follow-up will need to verify if this holds true for ablation lesions beyond the PVI. The average procedure time was 122.5 ± 42.85 min, similar to another focal PFA study [10], with slightly extended fluoroscopy time due to complex repeat procedures with additional lesions.

In our sample, many patients with multiple previous ablations underwent posterior box ablation to reduce atrial fibrillation burden by minimizing the triggering activity of the posterior wall [18]. Posterior box ablation using radiofrequency energy carries a risk of atrio-esophageal fistula, owing to its proximity to the esophagus [19]. Additionally, this procedure is time-consuming and often lacks long-term durability [20,21]. Given that PFA does not damage the esophagus [22], a reevaluation of the posterior box concept within the context of PFA is warranted. Reddy et al. demonstrated durable lesions in all cases after posterior box ablation using a single-shot PFA device [23], and Ruwald et al. showed that focal PFA was also highly effective on the posterior box [10]. This is in line with our study, where we successfully placed a posterior box in all applicable patients. Compared to data on radiofrequency ablation, where it showed no advantage over PVI alone [24], our results are promising, particularly because all our patients were discharged in sinus rhythm. This suggests that previous negative findings on the posterior box may be due to non-durable lesions in this area. Therefore, this concept could potentially prove successful, especially in repeat procedures. However, further randomized studies are desirable.

In 6.67% of patients, adenosine testing revealed dormant conduction, which was eliminated through repeated ablation. This finding differs from Ruwald et al.’s report of 100% negative adenosine tests in isolated PV [10]. This is crucial, considering the current lack of criteria for successful PFA ablation. Previously, the focus has primarily been on ablation settings such as energy setting, duration of energy delivery, and lesion tag diameter, focusing on overlapping tags [16]. Further strategies to ensure ablation lesion durability are necessary, with adenosine testing potentially being a valuable addition.

### 4.2. Sedation Concept

Left atrial ablation using PFA is commonly associated with discomforts like pain and coughing, affecting patient well-being and procedural accuracy [23]. Therefore, PFA is routinely performed under general anesthesia in some centers [10]. We, however, prefer deep sedation due to several advantages: it does not require an anesthesiologist, offering procedural flexibility; it often results in shorter procedure times due to faster sedation induction leading to more efficient use of operation room resources. This approach aligns with findings from percutaneous mitral valve repair and ERCP studies, which showed that general anesthesia led to longer procedure and sedation induction times without significantly improving patient satisfaction, compared to deep sedation [25,26].

We performed deep sedation using continuous propofol and remifentanil infusions. For significant agitation or coughing, we administered a single bolus of 12.5 mg ketamine. This approach helped avoid higher opiate doses and respiratory compromise. Sub-anesthetic ketamine (0.1–0.3 mg/kg) has been shown to decrease post-operative pain [27], agitation [28], and propofol requirements [29], with minimal side effects [30]. Recent guidelines recommend it as an opioid adjunct for perioperative pain [31]. Studies also report increased provider satisfaction and improved sedation quality when combining ketamine with propofol in emergency sedations [32]. In pediatric cardiac catheterization [33] and electrophysiological procedures in patients with hypotension and bradycardia, ketamine has proven effective [34]. A study demonstrated the safety and efficacy of higher dose ketamine (1 mg/kg) with midazolam and fentanyl for analgosedation, resulting in high patient and operator satisfaction [35]. However, in our practice, ketamine was used in lower, case-specific doses, required in 46.67% of cases, effectively enabling uninterrupted procedure continuation.

Using our sedation method, all procedures were conducted with an average nasal oxygen supply of 3.83 ± 1.56 L/min, without any cases of oxygen saturation drop requiring mask ventilation or intubation. Patients generally tolerated the procedure well under deep sedation, particularly following ketamine administration. In one case, a relevant 3D map shift occurred due to patient coughing during PFA application, requiring a remap.

While the necessity to supplement our initial deep sedation protocol with propofol and remifentanil with ketamine in some cases might be perceived as a limitation, it actually underscores our commitment to optimal analgosedation. This approach enabled us to precisely titrate the sedation to each patient’s needs, thereby achieving the best possible balance between minimizing discomfort and pain and maintaining cardiorespiratory stability. However, since coughing and patient movement under PFA can occur quite suddenly and ketamine has been effective in managing this with minimal side effects, we plan to adapt our protocol to include the routine administration of a low dose of ketamine before the ablation phase begins in future procedures.

The arterial blood gas (ABG) results mostly indicated moderate respiratory acidosis, which was without complications in all cases. This is in line with Aalbers et al., who found similar outcomes under deep sedation with propofol and remifentanil during PVI, recording a pCO_2_ of 53.48 ± 6.3 mmHg and pH of 7.29 ± 0.03. In their study, ABG levels returned to normal within 30 min post-procedure after stopping propofol, and hypercapnia did not adversely affect patients [36]. Fittingly, slightly elevated carbon dioxide levels also seem to be beneficial for heart and brain perfusion in patients after restoration of circulation [37,38]. PVI procedures using PFA generally need deeper sedation due to more intense pain and coughing compared to radiofrequency or cryoenergy ablation. Anić et al.’s research on PVI with focal PFA revealed that though general anesthesia offers more stable conditions, there is no significant difference in acute performance, safety, or chronic durability between deep sedation and general anesthesia [16].

### 4.3. Complications

In our study population, one major and one minor complication was observed. Specifically, during a CTI ablation using PFA, a patient developed vasospasm in the right coronary artery, as indicated by intermittent third-degree AV block and ST elevation. Symptoms completely resolved after administering intracoronary nitroglycerin in repeated, fractionated doses, and the patient had no symptoms during the 30-day follow-up. As vasospasm is a known risk when using PFA near coronary arteries, a prophylactic intravenous dose of 0.2 mg nitroglycerin was given before initiating ablation of the CTI [39]. Nonetheless, as indicated by this incident and supported by other studies on focal PFA [10], the prophylactic use of nitroglycerin might not always be sufficient, possibly due to suboptimal local concentration in the coronary vessels when administered intravenously. Consequently, using PFA near coronary arteries should be approached with significant caution, and considered only when there are no viable alternatives.

Vascular access complications, including post-procedural bleeding and groin hematoma, are known issues post-PVI, consistent with other studies [40]. These complications appear to be unrelated to sedation type. We believe this consistency is due to patient consciousness post-procedure. Awake patients can minimize movement, crucial for reducing vascular complications risk under pressure dressing following sheath removal.

We observed no sedation-related adverse effects, including hypoxemia requiring mask ventilation, consistent with a study on focal PFA under general anesthesia where no sedation-related issues like pneumonia or hemoptysis were noted. The low adverse outcome rate in our study, comparable to that in focal PFA with general anesthesia [10], reinforces our confidence in the safety of deep sedation for these procedures.

### 4.4. Limitations

Our study has some limitations. Firstly, the relatively small sample size limits generalizing our findings to a broader patient population. This was a deliberate choice due to the innovative nature of the procedure, where even initial insights are valuable. Secondly, while our acute success rates are promising, we need long-term data to evaluate lesion durability and atrial arrhythmia recurrence. We did not compare focal PFA with radiofrequency ablation at this point due to limited comparability because of the small sample size and the inhomogeneous cohort. Thirdly, the study focused on deep sedation without directly comparing it to general anesthesia, indicating an area for future research. Lastly, the efficacy of ketamine, though beneficial in our sample, requires further validation in randomized controlled trials.

## 5. Conclusions

In conclusion, our study provides initial insights into the feasibility, safety, and efficacy of complex left atrial ablation for arrhythmias under deep sedation using focal PFA. The procedure demonstrates good acute success rates, even among patients with a significant history of repeat ablations. The addition of ketamine to the propofol and remifentanil sedation regimen proved beneficial, especially when mapping was complicated by coughing or patient agitation during the procedure. These experiences have led us to plan an adaptation of our protocol, incorporating the routine administration of a low dose of ketamine prior to the ablation phase in future procedures. This adjustment aims to further enhance patient comfort and procedural stability. The observed dormant conduction, as indicated by adenosine testing, can help refine our understanding of the criteria for successful ablation with PFA. While these results are promising, larger studies are essential to determine the long-term efficacy of this approach.

## Figures and Tables

**Table 1:** Baseline clinical characteristics (n = 30).

Baseline Characteristics	n = 30
Age (years)	71.07 ± 7.11
Male	14 (46.47)
BMI (kg/m^2^)	27.95 ± 5.03
Type of arrhythmia - Paroxysmal AF - Persistent AF - Atrial flutter/tachycardia	3 (10) 22 (73.33) 17 (56.67)
CHA_2_DS_2_-VASc score	2.62 ± 1.21
LV ejection fraction (%)	56.64 ± 6.92
Moderate or severely dilated LA	18 (60)
Low voltage areas (<0.5 mV) in the LA (%)	34.09 ± 36.47
Chronic kidney disease	5 (16.67)
Coronary artery disease	6 (20)
Hypertension	24 (80)
Diabetes mellitus	1 (3.33)
Prior Stroke/TIA	0 (0)
Current Medication - Beta-blockers - Verapamil - Direct oral anticoagulant	22 (73.33) 2 (6.67) 30 (100)

Data given as n (%) or mean ± SD. Abbreviations: BMI, body mass index; AF, atrial fibrillation; LV, left ventricle; LA, left atrium; TIA, transient ischemic attack.

**Table 2:** Procedural parameters of ablation procedure.

	n = 30
Isolation/linear anatomical block	30 (100)
Patients discharged in sinus rhythm	30 (100)
Procedural duration (min)	122.5 ± 42.85
3D mapping system - CARTO (Biosense Webster) - Ensite X (Abbott Laboratories)	21 (70) 9 (30)
Fluoroscopy time (min)	9.14 ± 6.65
Dose area product (cGy*cm^2^)	654.3 ± 502.6
Rhythm at start of procedure - Sinus rhythm - Atrial fibrillation - Atypical flutter	15 (50) 12 (40) 3 (10)
First-time left atrial ablation - PVI only - Additional lesions	6 (20) 2 (33.33) 4 (66.67)
Repeat ablation - Re-PVI - Additional lesions	24 (80) 16 (66.67) 21 (87.5)
Posterior box	20 (66.67)
Anterior mitral line	15 (50)
Lateral mitral isthmus	2 (6.67)
Post-adenosine conduction recovery	2 (6.67)
CTI ablation	4 (13.33)
Major complication	0 (0%)
Minor complication	2 (6.67%)

Data given as n (%) or mean ± SD. Abbreviations: PVI pulmonary vein isolation; CTI, cavotricuspid isthmus.

**Table 3:** Sedation parameters of ablation procedure.

	n = 30
General anesthesia/intubation	0 (0)
Mean propofol infusion rate (mg/h)	286.3 ± 66.72
Mean remifentanil infusion rate (µg/h) RASS of −5 achieved before mapping	134.66 ± 45.76 30 (100)
Application of 12.5 mg ketamine	14 (46.67)
Nasal oxygen administered (L/min)	3.83 ± 1.56
Mapshift requiring remapping	1 (3.33)
Oxygen drop requiring mask ventilation	0 (0)
pH (before start of ablation)	7.28 ± 0.05
pCO_2_ (before start of ablation) (mmHg)	56.59 ± 8.29

Data given as n (%) or mean ± SD. RASS, Richmond Agitation-Sedation Scale.

**Table 4:** Complications in n = 30 cases.

Major Complications	
Procedure-related deaths	0 (0)
Atrio-esophageal fistulae	0 (0)
Procedure-related strokes or TIAs	0 (0)
Pericardial tamponades requiring intervention	0 (0)
Hemothorax	0 (0)
Severe air embolism with ST elevation	0 (0)
Nitrosensitive ST elevation (vasospasm)	1 (3.33)
New-onset renal failure requiring dialysis	0 (0)
Vascular access complications requiring intervention	0 (0)
**Minor Complications**	
Vascular access complications without intervention	1 (3.33)
Pneumonia	0 (0)
Hemoptysis	0 (0)
Acute hypersensitivity reactions to the contrast agent	0 (0)
Postinterventional pericarditis or small pericardial effusion	0 (0)
**Total Complications**	2 (6.67)

Data given as n (%). Abbreviations: TIA, transient ischemic attack.

## Data Availability

The data presented in this study are available on request from the corresponding authors.
